# Cooperation of Mtmr8 with PI3K Regulates Actin Filament Modeling and Muscle Development in Zebrafish

**DOI:** 10.1371/journal.pone.0004979

**Published:** 2009-03-26

**Authors:** Jie Mei, Zhi Li, Jian-Fang Gui

**Affiliations:** State Key Laboratory of Freshwater Ecology and Biotechnology, Institute of Hydrobiology, Chinese Academy of Sciences, Graduate School of Chinese Academy of Sciences, Wuhan, China; Ordway Research Institute, United States of America

## Abstract

**Background:**

It has been shown that mutations in at least four myotubularin family genes (*MTM1*, *MTMR1*, *2 and 13*) are causative for human neuromuscular disorders. However, the pathway and regulative mechanism remain unknown.

**Methodology/Principal Findings:**

Here, we reported a new role for Mtmr8 in neuromuscular development of zebrafish. Firstly, we cloned and characterized zebrafish *Mtmr8*, and revealed the expression pattern predominantly in the eye field and somites during early somitogenesis. Using morpholino knockdown, then, we observed that loss-of-function of Mtmr8 led to defects in somitogenesis. Subsequently, the possible underlying mechanism and signal pathway were examined. We first checked the Akt phosphorylation, and observed an increase of Akt phosphorylation in the morphant embryos. Furthermore, we studied the PH/G domain function within Mtmr8. Although the PH/G domain deletion by itself did not result in embryonic defect, addition of PI3K inhibitor LY294002 did give a defective phenotype in the PH/G deletion morphants, indicating that the PH/G domain was essential for *Mtmr8*'s function. Moreover, we investigated the cooperation of Mtmr8 with PI3K in actin filament modeling and muscle development, and found that both *Mtmr8*-MO1 and *Mtmr8*-MO2+LY294002 led to the disorganization of the actin cytoskeleton. In addition, we revealed a possible participation of Mtmr8 in the Hedgehog pathway, and cell transplantation experiments showed that Mtmr8 worked in a non-cell autonomous manner in actin modeling.

**Conclusion/Significance:**

The above data indicate that a conserved functional cooperation of Mtmr8 with PI3K regulates actin filament modeling and muscle development in zebrafish, and reveal a possible participation of Mtmr8 in the Hedgehog pathway. Therefore, this work provides a new clue to study the physiological function of MTM family members.

## Introduction

PTEN (phosphatase and tensin homolog deleted on chromosome ten) and MTM (myotubularin myopathy) family factors are members of the growing class of dual-specificity phosphatases (DSPs), which can dephosphorylate the products of phosphoinositide 3-kinase (PI3K) and antagonize downstream effectors using 3-phosphoinositides as ligands [1], [2]. They have been known to contribute to diverse processes that include cellular adhesion, signal transduction, and cell-cycle regulation[3]. Myotubularin-related (MTMR) phosphatases display a conserved active site motif CX5R, and an invariant sequence ‘CS**D**GW**D**R’ exists commonly in all the enzymatically active members[4]–[6]. Eight active members, including *MTMR8*, have been found in the MTM family. Several studies have further demonstrated that the PH/G domain functions to localize MTM to different subcellular compartments in the cell[7]–[9], and its deletion leads to activity loss of MTMR3 *in vitro* [10].

Mutations or altered expression of PTEN or MTM family members have been observed in human cancers[11]–[13] and in genetic development defects[14], [15]. Recent studies using RNA interference have revealed an unexpected role for several MTMs (including MTMR8) in promoting cell proliferation and survival [16], [17]. MTM1 is mutated in individuals with X-linked recessive myotubular myopathy [14]. MTM1 is adjacent to MTMR1 on the X chromosome, which plays a role in muscle formation and represents abnormal mRNA splicing in myotonic dystrophy [18]. Function loss of MTMR2 and mutation of MTMR13 causes Charcot–Marie–Tooth disease type 4B1 and 4B2[17], [19], a severe demyelinating neuropathy characterized by muscle weakness and sensory loss in the lower extremities beginning in early childhood. In addition, MTMR6 and MTMR9 were identified genetically to be required in *Caenorhabditis elegans* for endocytosis[20], [21]. Recently, the cell-therapy strategies, which use cultured myoblasts[22] or stem cells, have had notable successes in dystrophic mouse models[23] and DMD muscle[24]–[26], because transplanting whole cells have the potential to correct the symptoms of disease although each has its inherent disadvantages[27].

PI3K is conserved across eukaryotic organisms and regulates many facets of pathways involving cellular growth, survival, metabolism, vehicle trafficking, and chemotaxis[28]. The pathways controlling random movement parallel those of the amplification step of chemotaxis that is controlled through a regulatory loop containing Ras, PI3K, PTEN, and F-actin[29]. The initial activation of Ras and PI3K is independent of F-actin polymerization. However, F-actin polymerization is essential for amplifying the signal and stabilizing the leading edge in neutrophils and *Dictyostelium* [30], [31]. In *Dictyostelium*, F-actin recruits additional PI3K to the newly forming leading edge, enhancing the PIP3 response and downstream effector function [29], [30]. Cells with decreased PI3K activity exhibit a decrease in the second peak of RacB activation and F-actin polymerization, which has been linked to pseudopod extension.

To reveal the biological functions of MTM family members, we identified *Mtmr8* from the model animal zebrafish. Based on its expression pattern predominantly in the eye field and somites during early somitogenesis, we analyzed its physiological roles in embryo development by morpholino-mediated knockdown. We firstly showed that loss-of-function of *Mtmr8* led to defects in somitogenesis, and further examined the possible underlying mechanism and the PH/G domain function. Moreover, we investigated the cooperation of Mtmr8 with PI3K in actin filament modeling and muscle development, and revealed a possible participation of Mtmr8 in the Hedgehog pathway. The findings revealed a new role of Mtmr8 and its functional mechanism in neuromuscular development.

## Results

### Molecular characterization and expression pattern of *Mtmr8* in zebrafish embryos

**Figure 1:**
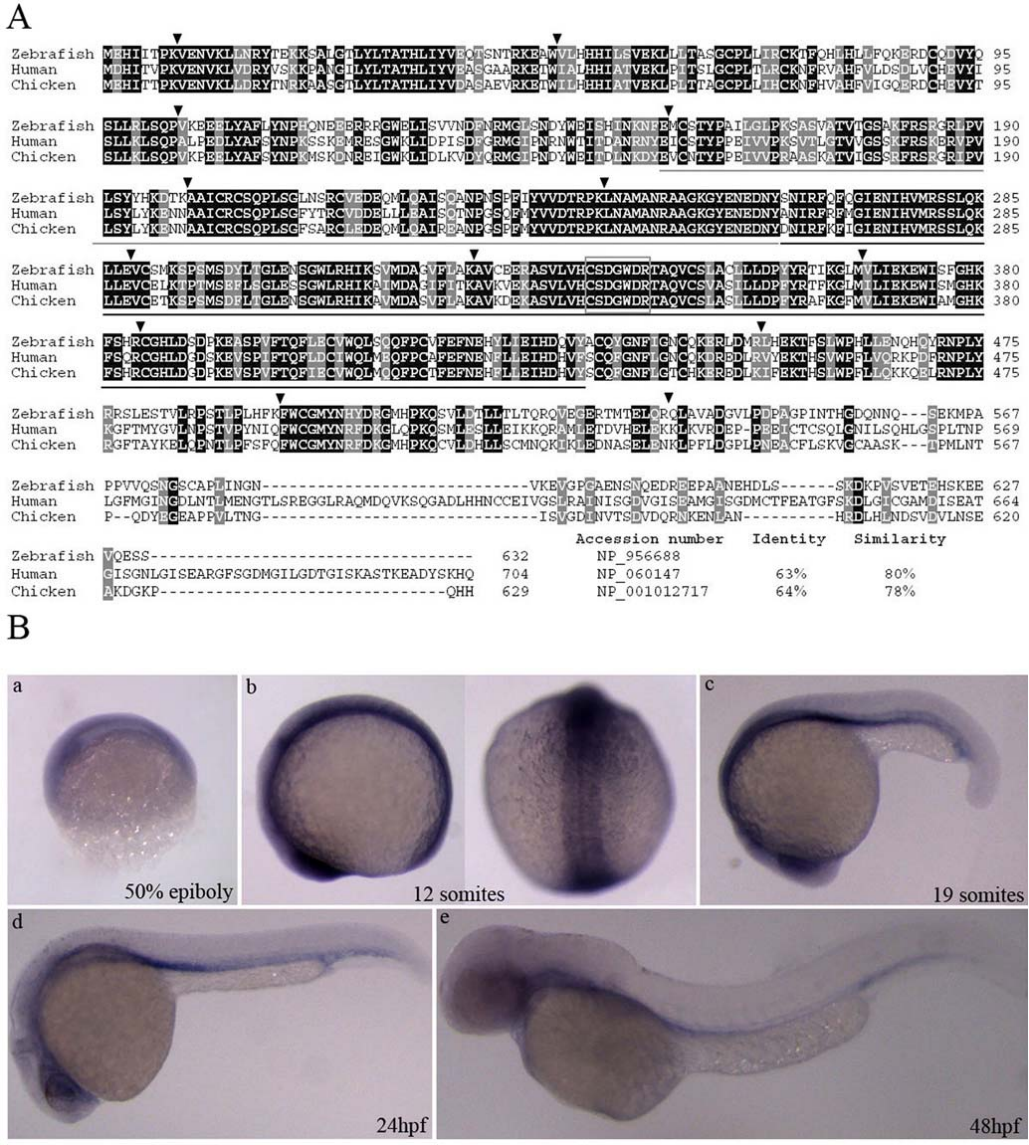
Sequence comparison and expression pattern of the deduced zebrafish Mtmr8. (A) Amino acid alignment of Mtmr8 between zebrafish with human and chicken. Similar and identical amino acids are highlighted in grey and black boxes. And the percentages of identities and similarities in *Mtmr8* were shown compared zebrafish with others. Arrowheads indicate the location of introns and are flanked by the corresponding exon numbers. The region encompassing the Myotub-related and PTPc_DSPc domain is underlined in grey and black lines. The rectangular box indicates the CX5R active site motif of enzymatically active members in the MTM family. (B) Expression pattern of zebrafish *Mtmr8*. Whole-mount RNA *in situ* hybridization were performed using a *Mtmr8* specific antisense riboprobe on embryos at the indicated stages. The arrows indicate the signals in the anterior and head. Embryos in panels are lateral view with the animal pole toward the top, and the right picture of panel b is dorsal view. The embryos in other panels are lateral views, with dorsal toward the top and anterior to the left. All scale bars are 100 µm.

The complete ORF for the *Mtmr8* encodes polypeptides of 632 amino acids, which contains 14 exons and 13 introns (identical to human MTMR8). An amino acid sequence alignment of zebrafish, chicken and human Mtmr8 polypeptides is shown in Fig. 1A. Zebrafish Mtmr8 exhibit 63% and 64% identity, and 80% and 78% similarity, with human and chicken Mtmr8 respectively, and higher identities exist in the Myotub-related and PTPc_DSPc motifs (amino acids 155–263 and 264–432 of zebrafish Mtmr8). The high homology implies that the zebrafish *Mtmr8* may have the same functions as in human.

Whole-mount *in situ* hybridization was used to analyze the expression pattern of *Mtmr8* during zebrafish embryogenesis. The expression distribution of *Mtmr8* was same to the result reported by Thisse B and C [32]. As shown in Fig. 1B, *Mtmr8* mRNA is expressed in prechordal plate and eye field at 50% epiboly (Fig. 1B-a). Between 1–13 somites, *Mtmr8* transcript becomes restricted to eye field and somites (Fig 1B-b). At 19 somites, *Mtmr8* expression is shown in the eye, telencephalon and ventral mesoderm (Fig 1B-c). At 24hpf, *Mtmr8* is expressed predominantly in the eye and vasculature (Fig 1B-d). Later, *Mtmr8* is expressed in the vasculature at 48hpf (Fig 1B-e).

### Targeted knockdown of zebrafish *Mtmr8* impaired embryo development

**Figure 2:**
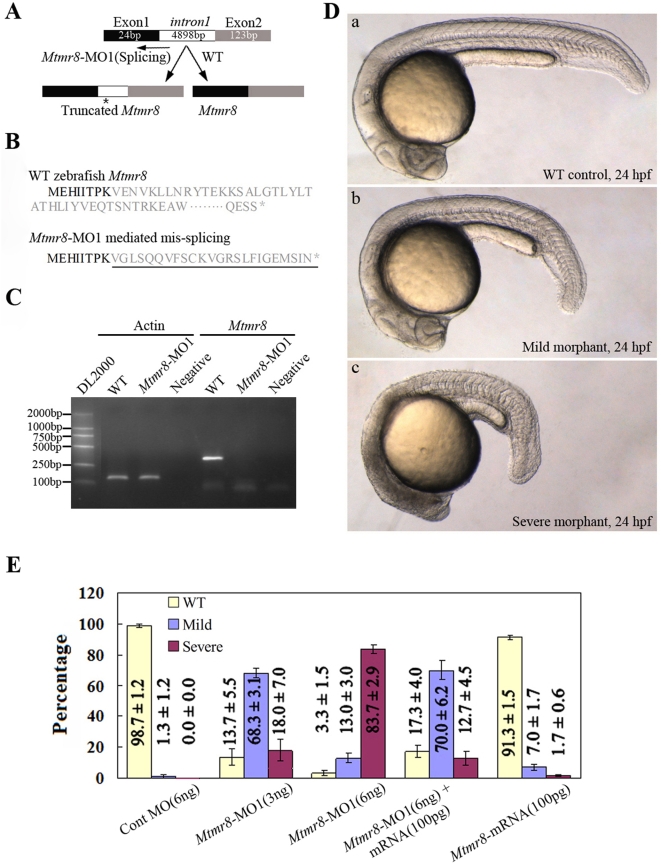
Targeted knockdown of *Mtmr8* using splicing morpholino in zebrafish embryos. (A) Diagram of splicing junction morpholino targeted against *Mtmr8* exon-intron boundary. (B) Amino acid sequences of wild type Mtmr8 and Mtmr8-MO1 mediated mis-splicing Mtmr8. Sequencing of the RT-PCR products revealed the mis-splicing transcript leading to a premature stop (asterisk) and causing a truncation in the protein (exon 1 in bold, the other exons in plain, and intron in plain and underlined). (C) RT-PCR detection of *Mtmr8* transcript at 24hpf in WT and *Mtmr8*-MO1 (6 ng) morpholino-injected embryos, comparing cryptic spliced transcript in the morpholino injected embryos to the cDNA PCR products. (D) Live morphology of WT control zebrafish embryo (a) and *Mtmr8*-MO1 knockdown embryo (b, c) at 24hpf. Injection volume was about 2 nL at 1-cell stage embryos. All scale bars are 100 µm. (E) Statistical data of three independent experiments on *Mtmr8* knockdown as well as its overexpression and *Mtmr8* mRNA rescue. Results are represented as mean±SD of three separate experiments (60 embryos in each experiment).

To determine the physiological effect of *Mtmr8*, we undertook loss-of-function experiments in zebrafish by using morpholinos. Firstly, we designed a splice junction morpholino targeted against the first coding exon-intron boundary to evaluate the knockdown effect and efficacy *in vivo*. As shown in Fig. 2A and Fig. 2B, the exon-intron morpholino (MO1) introduces intron 1 (about 5 kb) into the altered transcript, and includes a premature termination codon. Because the altered transcript is more than 5kb in its sequence, it could not be amplified from the morpholino-injected embryos in one minute by RT-PCR (Fig. 2C). In the control embryos, the amplified PCR product is 310 bp by the same primers designed from the first and third extrons (Fig. 2C). And, the altered transcript sequence was further amplified and verified by bi-directional DNA sequencing. Microscope observation indicated that the morpholino-mediated *Mtmr8* knockdown resulted in dramatic phenotypic abnormalities in somitogenesis (Fig 2D). At 24hpf, control experiments in which embryos were injected with Cont morpholino did not alter the wild-type phenotype throughout zebrafish development (Fig 2D-a), whereas the *Mtmr8*-MO1 morphants exhibited dose-dependent effect on embryos and resulted in mild and severe phenotype defects. In mild defects, morpholino-injected embryos showed delayed development, shorter and curled trunk and tail compared to wild-type or to control MO-injected embryos (Fig. 2D-b). In severe defects, the morphants were marked by small heads, abnormal tail fins, and U-shaped somites (Fig. 2D-c). Fig. 2E shows the percentages of normal, mild and severe defect embryos under different injection doses of morpholino. Moreover, an increasing percentage of normal or mild defect embryos was observed when the embryos were co-injected with 100 pg capped *Mtmr8* RNA and 6 ng *Mtmr8* MO1 (Fig. 2E). However, the defects could not be reduced when co-injected with 100 pg of capped GFP RNA and *Mtmr8* MO1 (Data not shown). In addition, overexpression of zebrafish *Mtmr8* by injection of capped RNA (100 pg) did not cause a visible phenotype. We used 100 pg doses in all the gain-of-function experiments described below if not indicated otherwise.

### Role of Mtmr8 in regulating PI3K/Akt signaling pathway

**Figure 3:**
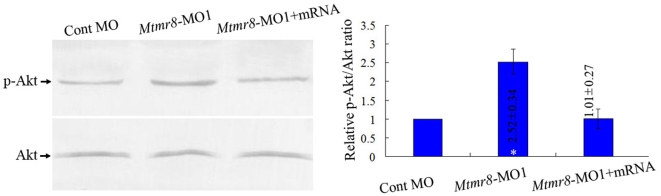
Phosphatase activity of zebrafish Mtmr8. Different extracts from Cont MO embryos, *Mtmr8* morphants and *Mtmr8* morphants co-injected with *Mtmr8* mRNA were prepared at 24 hpf (30 embryos/group) and subjected to Western blot detection with anti-Akt antibody or anti-phospho-Akt (pAkt) antibody. The corresponding histograms (*right panels*) plot the relative Ser(P)473-Akt to total Akt ratio (Relative p-Akt/Akt ratio), determined by band intensities that were analyzed by Scion software, with the ratio in experiment extracts normalized to the ratio determined in Cont MO injected embryo extracts. Results represent mean±SD of three separate experiments. *P<0.05.

Recent studies indicated that several MTMs might control PI3K/Akt activation by virtue of its ability to dephosphorylate PIP3[33]. However, there was no any direct report about the PIP3 lipid phosphatase activity of Mtmr8. Because knockdown of *Mtmr8* expression gave distinct developmental phenotypes, we asked whether the PIP3 lipid phosphatase activity of Mtmr8 was conserved in the development of zebrafish. To confirm it, we compared the level of phosphorylated Akt (pAkt) to total Akt (Akt) in whole fish lysates prepared from wild type zebrafish and *Mtmr8* morphant embryos. As shown in Fig 3, knockdown of *Mtmr8* expression produces a significant increase in the relative amount of pAkt compared to Cont MO injected embryos, whereas the elevated level of p-Akt could be reduced by coinjection with *Mtmr8* mRNA. These results indicate that zebrafish Mtmr8 exhibits PIP3 lipid phosphatase activity and functions to negatively regulate the PI3-Kinase/AKT pathway.

### Mtmr8 PH/G domain plays a critical role with PI3K in regulating zebrafish embryogenesis

**Figure 4:**
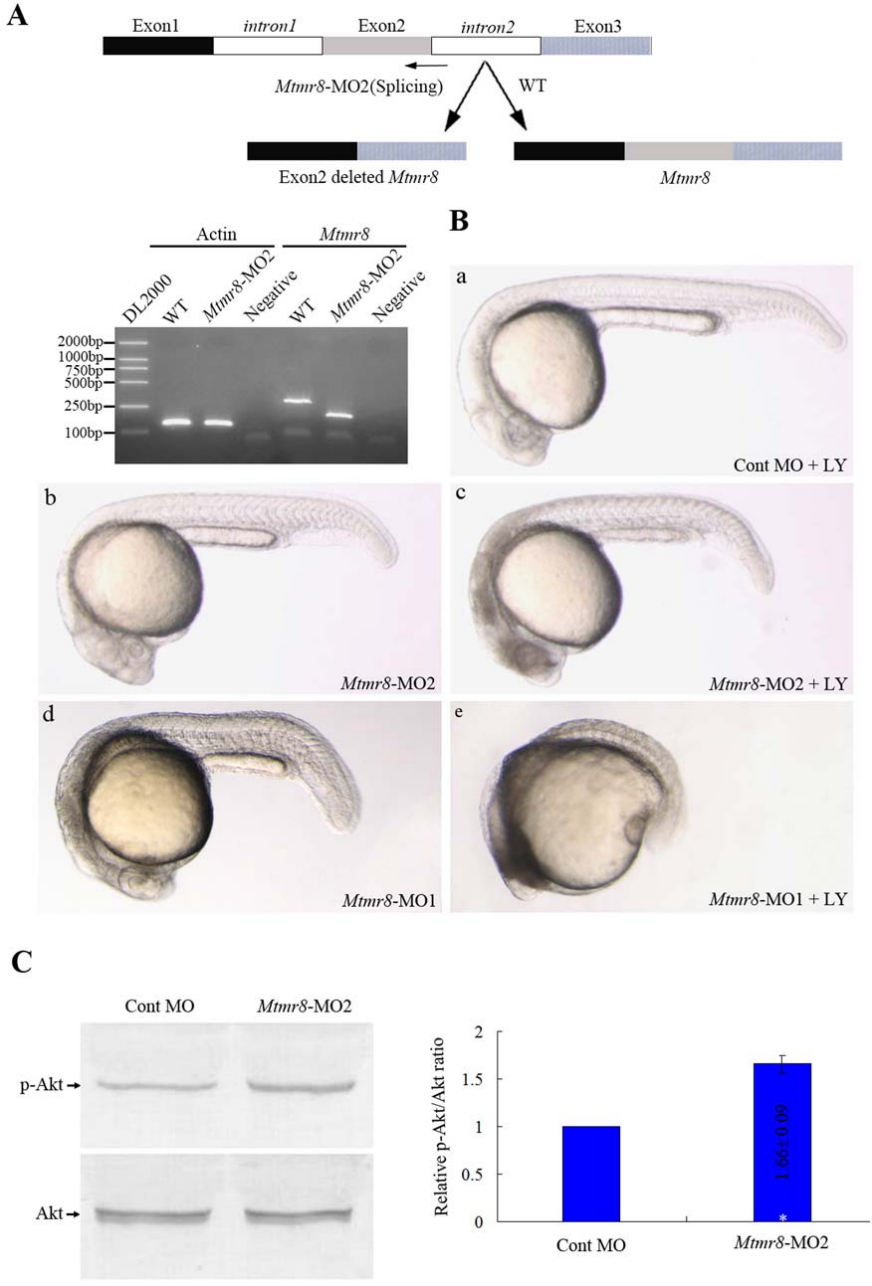
PH/G domain of *Mtmr8* is required to cooperate with PI3K regulating embryo development and essential for the phosphatase activity of zebrafish *Mtmr8* gene. (A) Splice junction morpholino targeted against *Mtmr8* exon-intron boundary resulted to loss of PH/G domain. RT-PCR of *Mtmr8* transcript at 24hpf in WT and *Mtmr8*-MO2 (6 ng) morpholino-injected embryos, comparing cryptic spliced transcript in the morpholino injected embryos to the cDNA PCR products. (B) Embryo phenotypes observed under the microscope. LY represents the PI3K inhibitor, LY294002. Each picture represents typical results out of three separate experiments (30 embryos in each experiment). All scale bars are 100 µm. (C) Immunoblot of different extracts from Cont MO embryos and *Mtmr8*-MO2 morphants at 24 hpf (30 embryos/group). Relative p-Akt/Akt ratio was determined by band intensities that were analyzed by Scion software, with the ratio in experiment extracts normalized to the ratio determined in Cont MO injected embryo extracts. Results represent mean±SD of three separate experiments. *P<0.05.

PH/G domain plays an important role in the activity of MTMR family [10]. To delete the PH/G domain of zebrafish *Mtmr8*, we designed a splice junction morpholino targeted against the second coding exon-intron boundary. In the *Mtmr8*-MO2 morphants, the targeted exon was eliminated, which encodes partial of the PH/G domain, as shown by a smaller PCR transcript which was verified by bi-directional DNA sequencing (Fig 4A). However, unlike *Mtmr8*-MO1 morphants (Fig 4B-a), the *Mtmr8*-MO2 morphants display no obvious defects, except mild abnormal in the head (Fig 4B-b). Intriguingly, previous studies showed that the defects resulted by homozygous single or double mutants of *ptena*−/− and *ptenb*−/−, were rescued by treatment with the phosphatidylinositol-3-kinase inhibitor, LY294002[34]. In order to assay the connections between Mtmr8 and PI3-Kinase/AKT pathway during embryo development, we inhibited the PI3K activity with 10 µM LY294002 between 10 hpf and 24 hpf, when transcript of *Mtmr8* became restricted to eye field and somites. When treated with 10 µM LY294002, the defects of *Mtmr8*-MO1 morphant embryos could be further deteriorated (Fig 4B-d), and the *Mtmr8*-MO2 morphants had severe defects (Fig 4B-e), comparing with Cont morpholino injected morphants which did not alter the wild-type phenotype throughout development 4B-c). In addition, loss of PH/G domain could also produce a significant increase in the relative amount of pAkt compared to Cont MO injected embryos (Fig 4C). The results indicate that the PH/G domain is essential for *Mtmr8*'s function and that protein encoded by the splice variant may lose part of its functions.

### Mtmr8 cooperation with PI3K regulates actin filament modeling

**Figure 5:**
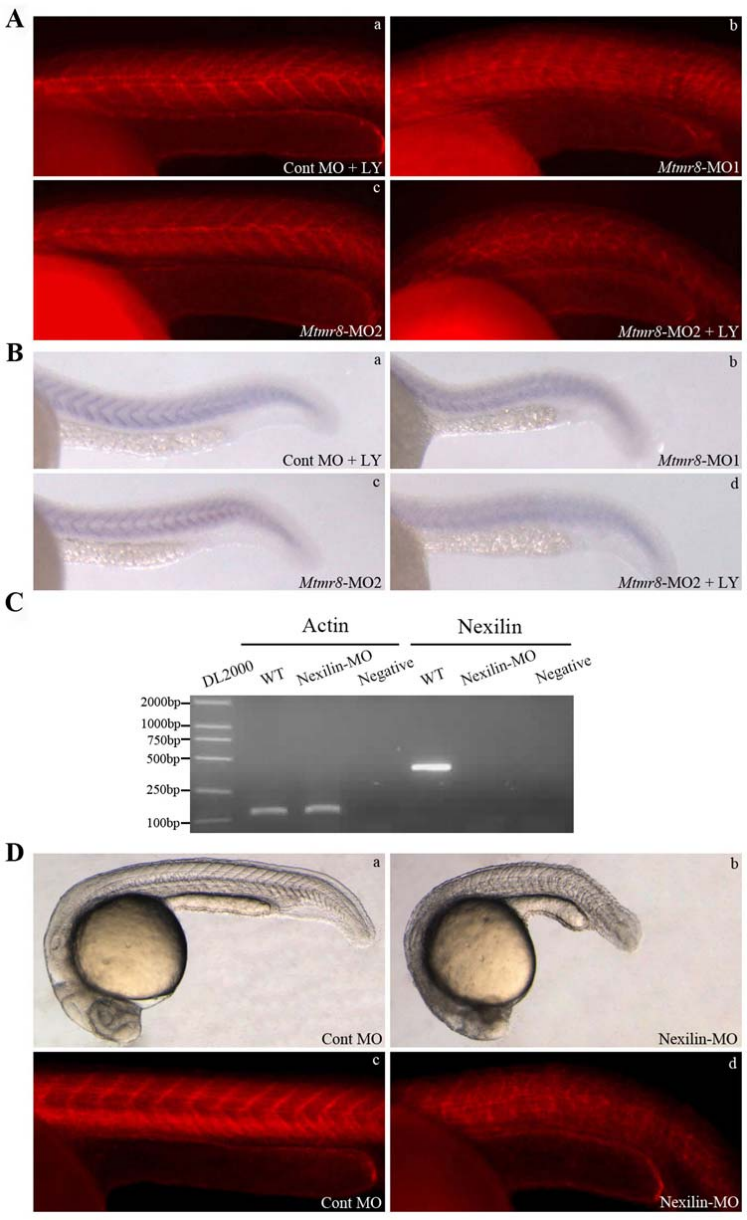
*Mtmr8* cooperates with PI3K regulating actin modeling. (A) Staining of embryos with phalloidin (F-actin) at 24hpf. (B) Nexilin mRNA expression at 24 hpf. (C) RT-PCR of *Nexilin* transcript at 24hpf in WT and *Nexilin*-MO (3 ng) morpholino-injected embryos, comparing cryptic spliced transcript in the morpholino injected embryos to the cDNA PCR products. (D) The effects of *Nexilin* knockdown on embryo development (a, b) and F-actin (c, d). The pictures are representative of at least three experiments (30 embryos in each experiment). All scale bars are 100 µm.

Mtmr8 is a member of the growing class of dual-specificity phosphatases (DSPs) as PTEN, which controls cell random movement through the Ras, PI3K, PTEN, and F-actin regulatory loop [29]. To examine a possible requirement for *Mtmr8* in integrity of the actin cytoskeleton, we used phalloidin-TRITC to stain filamentous actin in embryos at 24 hpf. In almost all the embryos treated with *Mtmr8*-MO1, we observed obvious defect of the actin cytoskeleton in a proportion of skeletal muscle fibres (Fig 5A-b), compared to the *Mtmr8*-MO2 morphant (Fig 5A-c) and control embryos (Fig 5A-a). However, when treated with LY294002, the actin cytoskeleton of *Mtmr8*-MO2 morphant was severely disturbed (Fig 5A-d).

Nexilin, a novel actin filament (F-actin)-binding protein, which was strongly expressed at somites at 24hpf [32]. We performed whole-mount *in situ* hybridization with zebrafish *Nexilin* riboprobes, and revealed that nexilin was significantly reduced and disorganized in the 24hpf *Mtmr8*-MO1 morphants (Fig 5B- b), However, the expression of nexilin was not obviously changed in *Mtmr8*-MO2 morphants (Fig 5B-c) and Cont MO morphants treated with LY294002 (Fig 5B-a). To further understand the function of Nexilin in integrity of the actin cytoskeleton, we performed morpholino to knockdown the zebrafish Nexilin. As shown by RT-PCR, in the control embryos, the amplified PCR product is 382 bp by primers designed from the first and second extrons. However, in the Nexilin-MO morphants, because of inclusion of intron 1, the product is longer than 3 kb, which could not be amplified in one minute (Fig 5C). Embryos injected with the 3 ng Nexilin MO and stained with phalloidin, did show severe skeletal muscle detachments (Fig 5d-b,d), compared to the control (Fig 5d-a,c).

### Mtmr8 is essential for the hedgehog pathway to regulate muscle development during embryogenesis

**Figure 6:**
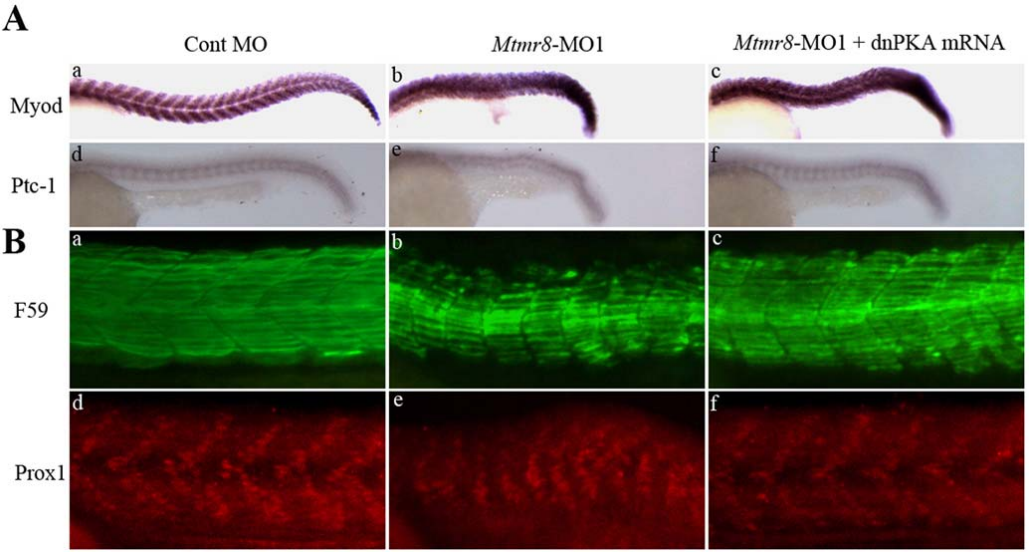
The effect of *Mtmr8* knockdown on hedgehog pathway and slow muscle development. (A) The expression of myod and ptc1 at 24hpf in the Cont MO (a, d), *Mtmr8*-MO1 morphant (b, e) and it injected with dnPKA mRNA (c, f). (B) Whole-mount staining of 24 hpf embryo with F59 antibody (green) and Prox1 antibody (red) and to reveal slow muscle cells. The pictures are representative of at least three experiments (30 embryos in each experiment). All scale bars are 100 µm.

The *Mtmr8* morphants have morphological traits in common with Hedgehog pathway mutants, such as U-shaped somite boundaries. To verify that the defects in *Mtmr8* morphants are correlated with the changes in Hh signaling, we examined the expression of *myod* and *patched1* (*ptc1*), a downstream target gene and receptor of Sonic hedgehog [35], [36]. Compared with the control (Fig 6A-a,d), *Myod* and *ptc1* expression is severely reduced and disorganized in *Mtmr8* morphants (Fig 6A-b,e). Dominant negative PKA (dnPKA) mRNA, when injected into embryos, results in a broader expression of Hh target genes and a rescue of the *Myod* and *ptc1* expression (Fig 6A-c,f). These results support the conclusion that *Mtmr8* is required for Hedgehog signaling in zebrafish development.

To study the function of Mtmr8 in slow muscle development, the embryos were then stained with antibody F59, which detects mostly slow myofibrils, although it also reacts weakly with fast myofibrils. While slow fibrils in Cont MO embryos were well organized with striation at 24 hpf (Fig 6B-a), fibrils of *Mtmr8* morpholino-injected embryos were shorter and reduced (Fig 6B-b). When injecting *Mtmr8* morpholino together with dnPKA mRNA, we detected myofibrils increased and longer than those of *Mtmr8* morphants (Fig 6B-c). Labeling with anti-Prox1, a slow muscle nuclear marker, shows that the numbers of muscle pioneer and slow muscle cells are reduced in *Mtmr8* morphant embryos (Fig 6B-e) compared to control embryos (Fig 6B-d), which is rescued by coinjection of dnPKA mRNA (Fig 6B-f). These findings indicate that the expression of *Mtmr8* is dependent on Hh signaling.

### Mtmr8 acts non-cell autonomously to F-actin modeling

**Figure 7:**
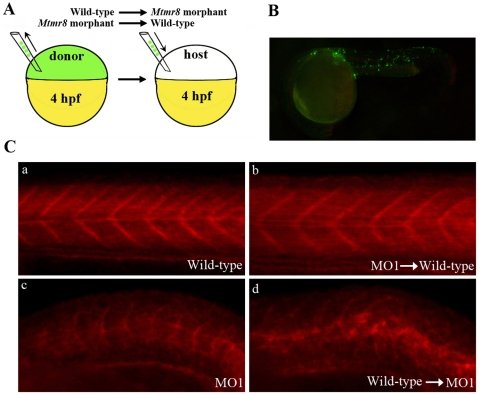
*Mtmr8* controls actin modeling non-cell-autonomously. (A) Schematic depiction of cell transplant experiments. (B) Representative picture of a chimeric embryo at 24 hpf. The labeling cells were mostly located in somites of trunk and tail regions. (C) The effects of cell transplantation on F-actin.

Previous studies have demonstrated that the transplanted labeled cells at the prospective mesodermal region (as shown in Fig 7A) will mainly differentiated into several regions including the neural tube, brain, and somites at 24 hpf when the muscle cells are easily distinguished from other cell types morphologically [37]. To determine whether *Mtmr8* acts in a cell autonomous or non-cell autonomous manner in muscle development, reciprocal cell transplantation experiments were carried out between wild-type and *Mtmr8* morphant embryos. We transplanted donor fluorescein-labeled cells into age-matched host embryos at 4 hpf. At 24 hpf, the phenotype of the embryos were observed under the microscope. The embryos with transplanted cell in the somites (Fig 7B) were chosen for phalloidin staining. In wild-type embryos, cells transplanted from *Mtmr8* morphant embryos could not change wild-type phenotype and F-actin modeling (Fig 7C-a, b, Table 1). In the reciprocal experiment, when wild-type donor cells were implanted into a *Mtmr8* morphant host, the phenotype was consistent with *Mtmr8* morphant, the U-shaped somites were still existed and the signal of F-actin was disorganized (Fig 7C-c, d, Table 1). We conclude that Mtmr8 protein acts non-cell autonomously within the actin assembly to promote slow fibres development.

**Table 1:** *Mtmr8* is non-cell autonomous for muscle F-actin modeling.

Donor genotype	Host genotype	Result
*Mtmr8* morphant	Wild-type	15/15 Wild-type (100%)
		10/10 Wild-type (100%)
		13/13 Wild-type (100%)
Wild-type	*Mtmr8* morphant	12/12 Severe defect (100%)
		13/13 Severe defect (100%)
		12/12 Severe defect (100%)

Donor embryos were labeled with fluorescein dextran. At 24 hpf, donor and host embryos were observed under the microscope, and then fixed for phalloidin labeling to determine genotype (as shown in Fig 7C). The phenotype of the transplanted embryos (number and percentage) is reported.

## Discussion

**Figure 8:**
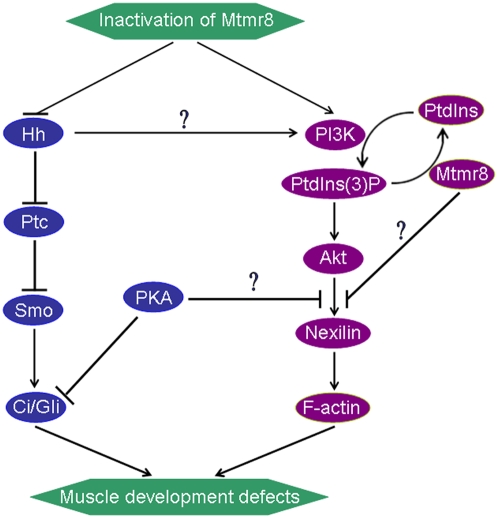
A hypothesized signal pathway that *Mtmr8* regulates zebrafish muscle development. Knockdown of *Mtmr8* promotes PI3K/Akt pathway and prevents Hh signaling pathway. The two pathways may interact with each other in regulating F-actin modeling and muscle development.

Recent studies clearly showed that myotubularin specifically dephosphorylates phosphatidylinositol 3-monophosphate (PI3P). The action of myotubularin on PI3P levels may implicate two parallel pathways by acting both as a protein phosphatase decreasing PI3P level by down-regulating PI3K activity and, a lipid phosphatase directly degrading PI3P than PI4P in vivo[38]. Such a dual activity has also been suggested for PTEN[39]. Mutations in MTMR family proteins are associated with the human neuromuscular disorders X-linked myotubular myopathy (myotubularin and MTMR1) and type 4B Charcot-Marie-Tooth neuropathy (MTMR2 and MTMR13). These diseases arise from impaired development and/or maturation of skeletal muscle cells and myelinating Schwann cells, respectively. Although a significant body of evidence has linked MTMR to endocytic membrane trafficking events, their role in neuromuscular development is currently unclear. Our findings in zebrafish demonstrated that Mtmr8 is an essential component, together with PI3K, nexilin and F-actin, of the positive-feedback cycle that maintains the normal development of muscle. According to previous studies[40]–[42] and our current studies, a diagram is depicted to explain Mtmr8 function and the relationship between Mtmr8 and the hedgehog pathway (Fig 8). Until now, because of no available means to prevent myotubular myopathy, our findings for the first time provided a new clue to study MTM family related disease *in vivo*.

In this study, we presented evidence that the PH/G domain is indeed a function domain of Mtmr8 that contributes to the development of zebrafish. Deletion of the PH/G domain could result in increasing the relative amount of pAkt (Fig. 4C). Akt is activated by various growth factors and hormones such as IGF-1 and insulin. Activation occurs at the plasma membrane after PI3K-dependent generation of PIP3. Thereby, Akt is recruited to the plasma membrane via its PH (pleckstrin homology) domain and is activated by phosphorylation at Thr^308^ by PDK1 followed by phosphorylation at Ser^473^ by the mTOR complex[43]. Previous studies have demonstrated that the PH/G domain in a number of different MTMs bind phosphoinositides and also function to localize MTMs to different subcellular compartments in the cell[7]–[9]. All these indicated that *Mtmr8* could inhibit PI3K/Akt activation through its functional PH/G domain, loss of which results in apoptotic signaling and cell death. Moreover, identifying the specific binding partners of the PH/G domains on the MTMs will provide important clues to the specific functions regulated by other MTMs as well as the mechanisms whereby loss of some MTMs lead to disease.

Recently studies demonstrated the existence of PI3K-dependent and -independent pathways for F-actin polymerization during chemoattractant-stimulated lamella extension in the human neutrophil. One pathway is dependent on PI3Kγ activation and downstream is dependent on PKCδ and Akt/PKB. This pathway controls the formation of 70% to 80% of the F-actin in the lamella region [44]. *In vitro* studies, activation of PI3K activity alone is sufficient to remodel actin filaments to increase cell migration through the activation of Akt and p70S6K1 in CEF cells [45]. However, *in vivo*, the F-actin polymerization and modeling is also *Mtmr8*-dependent. Although the PI3K/Akt was activated in *Mtmr8* knocked embryos, it was not enough to model actin filaments alone. *Nexilin* is a F-actin binding protein and mediates cell motility, over-expression of which promoted cell migration and adhesion[46]. However, the mechanism of its function is not clear. In *Mtmr8* deficient embryos, the expression of *Nexilin* was reduced, which induced the defect of F-actin modeling. Inhibition of PI3K with LY294002 did not alter the initial formation of these F-actin-rich cup structures at the plasma membrane but it did prevent Akt/PKB recruitment to these cups and their subsequent fusion into the large rings characteristic of normal[47]. However, the effects of inhibiting PI3K on the embryo cytoskeleton are not well characterized. Low dose of LY294002 didn't obviously affect F-actin, whereas it severely disturbed the modeling of F-actin in *Mtmr8* morphant and PH/G losing embryos. These indicated that the increasing of pAkt is a very important recovery mechanism for *Mtmr8* deficient, although it could not completely rescue the defects.

The model about the mechanisms underlying the Hh signalling pathway is clearly shown by Masai *et al.* [41]. And Sonic hedgehog (Shh) has been reported to act as a mitogen and survival factor for muscle satellite cells. PI3K/Akt pathway is essential for Shh's actions and directly involved in adult muscle cell proliferation and differentiation. Shh induces Akt phosphorylation in adult muscle cells and influences the PI3K/Akt pathways in a manner similar to IGF-I [48]. Although an increase in Akt phosphorylation could be detected in response to knockdown of *Mtmr8*, it was not enough to rescue the expression of *Myod* and *Ptc1*. Over-expression of dnPKA mRNA, a downstream of Shh, could reverse the defect induced by *Mtmr8* deficient. Previous findings provide a basis for the synergistic role of PI3-kinase/Akt in Hh signaling in embryonic development and Hh-dependent tumors [49]. The PI3K inhibitor LY294002 inhibited migrating neo cells and was able to further inhibit residual dnPKA cell migration. PKA may play an important role in the signaling processes that lead to motility. Either inhibition or hyper-activation of PKA may inhibit cell migration, F-actin polymerization and synthesis [42], [50]–[52]. Knockdown of *Mtmr8* led to hyper-activation of PKA, which caused the reduction expression of *Nexilin* and F-actin. All these suggested that *Mtmr8* and PI3K/Akt play a synergistic role in regulation of Hh signaling in embryonic muscle development.

Currently, many potential therapies are evaluated by introducing fluorescently tagged cells into a diseased animal and then following their fate using fluorescence to determine if the introduced cells localize to muscle and participate in the repair process[27]. Mutation or knockdown of several genes in zebrafish have been shown to be the underlying basis of many muscular defects[53]–[56]. Cell transplantation experiments could confirm that the gene function is required cell-autonomously or non-cell-autonomously within the muscle development, which may be as a model to evaluate the possibility to use cell therapy for human genetic muscle disease during early fetus. *Mtmr8* acts in non-cell-autonomous manner during embryos muscle development, which may suggest that cell therapy is not an efficient way to rescue the defect during early embryo development. The effectiveness of cell therapy could also be evaluated in human neuromuscular disorders caused by mutations in other myotubularin family genes.

From our studies, we have demonstrated that Mtmr8 have the same function as lipid phosphatases PTEN to dephosphorylate the PI3K products by down-regulating PI3K activity, and to regulate actin modeling and muscle development of zebrafish [57]. However, it is different from tumor suppressor gene, PTEN. Mtmr8 is not only an antagonist of PI3K, but also as a partner to balance the PI3K expression in embryo development regulation of zebrafish.

## Materials and Methods

### Maintenance of zebrafish

A breeding colony of zebrafish (*Danio rerio*) were maintained at 28.5°C on a 14 h (hour) light/10 h dark cycle [58]. All embryos were collected by natural spawning and staged according to Kimmel et al. [59]. Kinase inhibitors, LY294002 were dissolved in DMSO at stock concentration of 50 mM, and then diluted to final concentration of 10 µM in embryo media from 10 hpf to 24 hpf, which had no obvious effect on embryos survival, activity and health. Control embryos were treated with the equivalent amount of DMSO solution.

### RT-PCR and Western blotting

Total RNAs were isolated by SV RNA Isolation Reagent (Promega) from different stages and tissues, and the concentration and quality were determined by agarose electrophoresis and spectrophotometer. After treating with DNase I (RNase-free, Promega), the RNAs (about 1 µg) were reverse-transcribed with MMLV (Gibco) at 37°C with oligo(dT)15 primer. *β-actin* was used as internal control genes. All samples were analyzed in triplicates. Phosphatase activity of zebrafish Mtmr8 and Western blot detection were performed according to previous reports[60], [61]. Images of blots were captured with a scanner, and quantitative densitometric analysis was performed using Scion Image.

### Antisense morpholino and mRNA microinjection

Morpholinos were synthesized by GeneTools LLC (Philomath, OR). Following are the sequences for various morpholinos: *Mtmr8*-MO1 (Splicing antisense), 5′-CACCTGCTGACTCAGACCTACCTTC-3′; Mtmr8-MO2 (Splicing antisense), 5′-GGCCAACATTACCCATGTTTCTTTG-3′; Nexilin-MO (Splicing antisense), 5′-ATAGCCTCTTCATACTTTACCTCTT-3′; Standard control MO (Cont MO), 5′-CCTCTTACCTCAGTTACAATTTATA-3′. For injection, MOs were injected into fertilized zebrafish eggs at the one-cell stage at a concentration of about 6 ng each embryo. After injection, embryos were incubated at 28.5°C in Embryo Medium [58].

Plasmids pCS2-*Mtmr8* and pCS2-*dnPKA* were linearized for in vitro transcription. Capped sense RNAs were synthesized using SP6 RNA polymerase and the SP6 Cap-Scribe (Roche), following the manufacturer's instructions, re-suspended in water and injected at a concentration of 100 ng/µL.

### Whole-mount in situ hybridization and immunohistochemistry

Embryos at different stages were collected and pre-treated and fixed as described [57]. Purified plasmids was linearized by selected restriction enzymes and used as templates for *in vitro* transcription using T7 or Sp6 RNA polymerase to generate DIG-labeled (Roche) sense and anti-sense probes. *In situ* hybridization was performed as described [62].

F59 antibody was purchased from Developmental Studies Hybridoma Bank (DSHB, University of *Iowa*, USA), and Prox1 antibody from Abcam (Cambridge, United Kingdom). Zebrafish embryos were fixed overnight in 4% paraformadehyde at 4°C, and then washed in 0.1% Triton in PBS (PBT) and dechorionated. They were then incubated for 1 h in 0.5% Triton in PBS, followed by 5-h incubation in block solution (10% normal goat serum, 1% DMSO, 0.1% Triton in PBS). Embryos were then incubated overnight at 4°C in block solution containing Phalloidin and/or primary antibodies. They were then washed in PBT, and incubated for 5 h at room temperature with secondary antibodies. Antibody and Phalloidin staining of zebrafish embryos were performed as previously described [63].

### Transplantation

Embryos, which were used as donors at later stages, were injected between one-cell to two-cell stages with 2.5% fluorescein dextran (Molecular Probes product) alone or in combination with 6 ng morpholino. Donor and host embryos were dechorionated. The cells were sucked from the prospective mesodermal region of a donor at the sphere stage, and transplanted into the same position of wild-type host embryos at the same stage. Locations of transplanted cells in the hosts were determined by fluorescence stereomicroscopy at the shield stage. Distribution of transplanted cells in the hosts were observed and photographed at 24 hpf postfertilization (hpf) using a Leica fluorescence stereomicroscope. We sucked out the embryos with transplanted cell mainly in the somites, which were fixed at 24 hpf for 2 h at room temperature in 4% paraformaldehyde, and later for phalloidin staining.

### Statistical analysis

Data are presented as mean±SD. The relative expression level of p-Akt in *Mtmr8* morphants and control morpholino groups were compared statistically using one-way analysis of variance (ANOVA), followed by the Tukey's post hoc test for multiple comparisons. A probability (P) of <0.05 was statistically considered significant.
